# Ticks: Biology, Habitat, Threats and Protection Methods

**DOI:** 10.3390/biology15060497

**Published:** 2026-03-20

**Authors:** Marlena Szalata, Karolina Wielgus, Mikołaj Danielewski, Andrzej Hnatyszyn, Milena Szalata, Marzena Skrzypczak-Zielińska, Ryszard Słomski

**Affiliations:** 1 Department of Biochemistry and Biotechnology, Poznań University of Life Sciences, Dojazd 11, 60-632 Poznań, Poland; marlena.szalata@up.poznan.pl; 2 Department of Pediatric Gastroenterology and Metabolic Diseases, Poznań University of Medical Sciences, Szpitalna 27/33, 60-572 Poznań, Poland; kwielgus@ump.edu.pl (K.W.); danielewski.mikolaj@gmail.com (M.D.); 3 Independent Public Health Care Centre in Nowa Sól, Multispecialty Hospital, Chałubińskiego 7, 67-100 Nowa Sól, Poland; andrzej.hnatyszyn@gmail.com; 4 Department of Biotechnology, Institute of Natural Fibres and Medicinal Plants-National Research Institute, Wojska Polskiego 71B, 60-630 Poznań, Poland; milena.szalata@iwnirz.pl; 5 Institute of Human Genetics, Polish Academy of Sciences, Strzeszyńska 32, 60-479 Poznań, Poland; marzena.skrzypczak-zielinska@igcz.poznan.pl; 6 Institute of Medical Sciences, College of Social and Media Culture in Toruń, św. Józefa 23/35, 87-100 Toruń, Poland

**Keywords:** *Ixodes* sp., life cycle, host, habitat, tick-borne diseases, Lyme disease, encephalitis, prevention methods

## Abstract

The most common species of tick in Europe is the castor bean tick (*Ixodes ricinus*), which is found in forests, parks, and gardens and is active almost all year round. Ticks are among the most important arthropods and vectors of disease, transmitting a wide range of parasites that sometimes lead to the death of infected organisms. The peak incidence of tick-borne diseases occurs between May and September; however, due to global warming, people are increasingly exposed to tick-borne diseases throughout the year. In order to increase the possibility of preventing the transmission of diseases by ticks, it is necessary to become thoroughly familiar with the life cycle of ticks and the environment in which they live. Vaccines are available for some diseases, such as tick-borne encephalitis, while others require a highly specific diagnosis. Another major problem is the long period between the tick bite, which often goes unnoticed or is even ignored by the patient or the doctor, and the development of tick-borne diseases. Increasing attention is being paid to the prevention of tick-borne diseases through prevention of tick bites, quick tick removal, use of repellents, appropriate land management, vaccinations, and the use of plants as natural acaricides.

## Simple Summary

Recently, increasing attention has been paid to tick bites and the diseases they transmit. Ticks have always accompanied humans and animals, but they can now move rapidly and expand their feeding range. Ticks from the *Ixodes* family are found in many parts of the world, except for mountainous regions. Ticks transmit Lyme disease, tick-borne encephalitis, anaplasmosis, babesiosis, Powassan infection, and other diseases to humans and animals. They are active from March to November, practically year-round, due to global warming. Hosts include birds, lizards, hedgehogs, goats, roe deer, fallow deer, red deer, foxes, dogs, cats, and humans. The tick genome has already been identified in 10-year surveillance studies, which is crucial for interpreting gene structure. Approximately 70% of the genome consists of repetitive, often mutated, genes. They comprise single copies of the same genus that differ from others to aid arachnid survival. Researchers are examining both genes and applications that facilitate the use and transmission of diseases, potentially helping to develop new treatments and vaccines. Unique aspects of tick biology have been identified. For instance, their saliva contains various bactericidal, analgesic, anticoagulant, and immunosuppressive substances, which may cause ticks to suck blood unnoticed. Ticks also contain enzymes that may possess insecticides.

## 1. Introduction

Recently, much attention has been paid to ticks and the diseases they carry; however, there is a lack of public understanding and dissemination of expertise in this field. Ticks have always coexisted alongside humans and animals, but now, it is possible for them to travel quickly and efficiently through planes. The aim of this study is to present basic information on the life cycle, biology, genetics, and habitats of the most common ticks; the diseases they transmit; ways to prevent risks through contact prevention, rapid removal of ticks from the body, appropriate land management including in urban green areas, and vaccinations; and challenges related to the diagnosis of tick-borne diseases. Much attention has been devoted to the possibility of using plants as natural acaricides. This study mainly focuses on Europe and the main tick species, *Ixodes ricinus* (Linnaeus 1758). One section of this study focuses on the increased tick contact that professional groups who stay in forested areas have. As such, soldiers and foresters are at increased risk of tick exposure.

The most common tick species in Europe is *Ixodes ricinus*. The word *ricinus* refers to its resemblance to castor bean seeds. These ticks are common in Europe, and their presence in the mountains is increasing due to global warming. Ticks may transmit Lyme disease, encephalitis virus, and other diseases to humans and animals [1,2]. In the United States, the primary tick species that transmits Lyme disease is the deer tick *Ixodes scapularis* (Say 1821), found in the northeastern, mid-Atlantic, and northern-midwestern parts of the country [3]. It is interesting that there are differences in the severity of Lyme disease depending on ethnic background; this may also be related to behavioral and environmental factors, such as more frequent exposure to ticks and access to appropriate diagnostics [4].

Tick nymphs, females, and males are usually active from March to November, but due to the effects of global warming, they are now practically active year-round. Ticks can be found in many places besides forests, including parks, gardens, meadows, dunes, and even backyards, preferring grassy, bushy areas and leaf litter where they wait for animal or human hosts [5]. Adult females reach a size of 3–4 mm, but after feeding, they expand to 10 mm. Adult males measure up to 3.5 mm. Other tick developmental stages also pose dangers; nymphs measure approximately 1.5 mm before feeding, and larvae measure approximately 1 mm. Hosts include birds, lizards, hedgehogs, goats, roe deer, fallow deer, red deer, foxes, dogs, cats, and humans [6].

Below, we present information on tick biology, habitats, and genomes, as well as factors influencing tick exposure. A summary is presented of the main diseases transmitted by ticks as vectors, as well as the possibilities of protection against ticks, with particular attention paid to Europe.

## 2. Methods

Information relevant to the biology, habitat, and threats of ticks, as well as protective methods, was collected by searching scientific databases, including Google Scholar, PubMed, ScienceDirect, and Springer. For epidemiological data and tick species health, the following databases were used: The Food and Agriculture Organization of the United Nations, the European Centre for Disease Prevention and Control, and the U.S. Centers for Disease Control and Prevention. The following national health agencies were also consulted: UK Health Security Agency, EpiCentro–Epidemiology for public health, and National Institute of Public Health, NIH–National Research Institute. We used information presented by scientific societies within scope of epidemiology or monitoring of tick species. The selection of materials was based on the names of ticks, their biology, where they occur, their importance as disease vectors, selected diseases, and various methods of prevention. Due to the use of data available on the Internet, as well as peer-reviewed articles, the selection was based on scientifically confirmed data. Due to the participation of one of the authors (RS) in the work of the Biological Weapon Convention during the presentation of research at the Military Institute of Microbiology in Munich, Germany, where attention was drawn to the importance of detecting and preventing tick-borne diseases, further interest in this topic was developed.

## 3. Tick Biology

Ticks are arachnids belonging to the order *Ixodida* are divided into three families: *Ixodidae* (Koch 1844) representing hard ticks and *Argasidae* (Koch 1844) representing soft ticks. The third family *Nuttalliellidae* (Schulze 1935) encompasses only one genus *Nuttalliella* (Bedford 1931). The taxonomy of ticks, number of species and their nomenclature still needs proper evaluation [7,8]. They are specialized, obligate, nonpermanent external parasites that feed on the blood, lymph, or digested tissues of mammals, birds, reptiles, and amphibians in all regions of the world [9].

The life cycle of ticks is influenced by temperature, the length of day, and blood consumption; individual females can lay thousands of eggs. Their external anatomy consists of a capitulum (gnathosoma) strictly connected with the body (idiosoma) and legs, as well as a hypostome, which enables the feeding process. There are differences in the number of legs depending on the stage of development: six legs in the larval stage, and eight legs in the nymph and adult stages of ticks. The lifespan of ticks can range from 2 to 6 years for hard ticks of the genus *Ixodidae* and up to 20 years for soft ticks, with most of this time spent away from the host. The transition to subsequent stages of development requires blood consumption, during which ticks can transmit various pathogens to the host; hence, much attention has been paid to thoroughly understanding the mechanisms of feeding. *Ixodes ricinus* attaches itself to its host using its mouthparts, and the proteins it secretes form an additional adhesive layer around the attachment site. While feeding, the tick thickens its food, periodically removing excess water and salt back into the host through its salivary glands. There are two or three feeding phases: the preparatory phase, the slow feeding phase, and the rapid feeding phase. Throughout, the tick’s body increases significantly in size. This phase is associated with the transmission of pathogens to the host. At the end of feeding, the tick withdraws its mouthparts and detaches from the host [10,11]. It is assumed that ticks can feed intermittently, which necessitates the use of multiple hosts to complete a given stage of development [9,12].

Appetite-inducing stimuli include odor, vibration, shadowing, and visual appearance. The feeding of hard *Ixodidae* ticks is slowed by the production of a cuticle, and females can increase their mass over 100-fold. Soft ticks, on the other hand, feed almost immediately after attachment, shortening the feeding time, depending on the developmental stage, from 20 min (larvae) to 70 min (adults). Furthermore, they do not secrete cement or new cuticles, and they do not regurgitate excess water back into the host but rather secrete it through their hip pores.

Pheromones play a crucial role in the tick life cycle. Among these, three groups are worth noting: assembly pheromones, aggregation and attachment pheromones, and sex pheromones. Assembly pheromones attract ticks to favorable habitats, facilitating the finding of hosts and contact with other ticks of a given species, ensuring population survival. Aggregation and attachment pheromones are secreted by males feeding on the host, attracting females of the same species (e.g., *Amblyomma* spp., Koch 1844). Sex pheromones secreted by feeding females attract males, facilitating copulation [13].

The life cycle of *Argasidae* ticks includes an egg, a six-legged larva, eight-legged nymphs (for up to eight larval stages), and eight-legged adult males and females. The developmental stages of *Ixodidae* ticks include an egg, a six-legged larva, a single eight-legged nymph stage, and eight-legged adult males and females. In most cases, progression to the next developmental stage and reproduction requires a blood meal. Interestingly, pathogens present in one tick developmental stage are passed on to subsequent stages—this is known as transstadial transmission. The development of specific developmental stages can be influenced by geographic location, host relationships, and environmental conditions, such as the number of hours of daylight to which the ticks are exposed and humidity.

Unfed *I. ricinus* ticks (nymphs and adults) begin searching for a host (questing) in spring at temperatures of about 3–10 °C in the absence of snow cover. These processes are also regulated by ambient humidity and the tick’s hydration level, which determines the maximum temperature at which the tick can function; hence, the maximum temperature limit of 35 °C is not always reached. The main peak of activity for *I. ricinus* nymphs and adults in Central and northern Europe is observed from April to July, with an additional smaller peak occurring in autumn. In the case of tick larvae, the first peak occurs in May, but the second peak in mid-summer precedes the autumn peak of activity of adults and nymphs [11]. The occurrence of unfavorable conditions during the lifespan and development of ticks is associated with the occurrence of diapause, which is a physiological reaction to external stimuli, shortening the time spent on developmental and behavioral activity [14].

Ticks play an important role in the environment, serving as food for many insects (e.g., ants and some beetles), amphibians (frogs), reptiles (lizards and snakes), birds (e.g., guinea fowl, wild turkeys, chickens, oxpeckers, and some songbirds), and mammals (e.g., rodents, hedgehogs, squirrels, and shrews). This naturally controls tick populations. It can be assumed that ticks are one of many factors controlling wild animal populations, and contact with domestic animals also helps ticks gain new habitats. The choice of hosts by ticks of the genus *Ixodes* seems to result from ecological conditions, assuming that ticks and hosts coexist in the same abiotic environment. This can be confirmed by ticks using a wide range of hosts at different stages of development, in most cases without co-phylogenetic events with specific hosts [15]. By hunting animals and potentially transmitting diseases, they naturally eliminate weaker and less adapted individuals, improving the overall health of the species. Ticks are a natural form of population control for certain animal species, preventing overpopulation and ensuring the preservation of biodiversity. They also ensure a healthy balance in the environment; however, they can also cause the extinction of host species weakened by the coexistence of adverse environmental factors and excessive hunting, as in the case of the South American marsh deer (*Blastocerus dichotomus*, Illiger 1815) [16]. Hypothetically, it can be assumed that ticks participate in the circulation of nutrients in the environment; while feeding, they absorb nutrients, which, together with metabolic products, return to the soil, increasing the availability of appropriate nutrients for plants and other organisms. There are reports of the possibility of using them as bioindicators of environmental pollution [17].

The larva and nymph of the *I. ricinus* tick feed on over 300 species of vertebrates, mainly mammals like the wood mouse *Apodemus sylvaticus* (Linnaeus 1758) and birds such as passerines and reptiles. Interestingly, ticks at immature stages are incapable of attaching to red foxes, *Vulpes vulpes* (Linnaeus 1758), dogs, and some other predators. The adult tick feeds exclusively on medium- and large-sized mammals, such as hedgehogs *Erinaceus europaeus* (Linnaeus 1758) and *Erinaceus roumanicus* (Barrett-Hamilton 1900), and cervids such as roe deer *Capreolus capreolus* (Linnaeus 1758), European fallow deer *Dama dama* (Linnaeus 1758), and red deer *Cervus elaphus* (Linnaeus 1758); ticks are also found on wild boar *Sus scrofa* (Linnaeus 1758). In the absence of deer, wild rabbits *Oryctolagus cuniculus* (Linnaeus 1758) and hares, e.g., the European brown hare *Lepus europaeus* (Pallas 1778) become the main host, and in agricultural environments, sheep and cattle are targeted [18,19,20]. Tick abundance often correlates with high densities of smaller mammals, such as rats, squirrels, and rabbits. Declining tick populations may indicate that predators of smaller animals are getting out of control. Three main diseases are believed to contribute to wildlife population control: borreliosis and different rickettsiosis, e.g., Rocky Mountain spotted fever, and tularemia, which are transmitted by ticks [21]. The effect of rodent population size on *Ixodes ricinus* and *Ixodes scapularis* nymph populations has also been observed; for instance, there is an increase 2–3 years after the baby boom in the rodent population, which hosts the larvae. In the case of cervids, the presence of the animals themselves seems to be important, rather than their density, because they can be hosts for any development stage of ticks [11].

**Figure 1:**
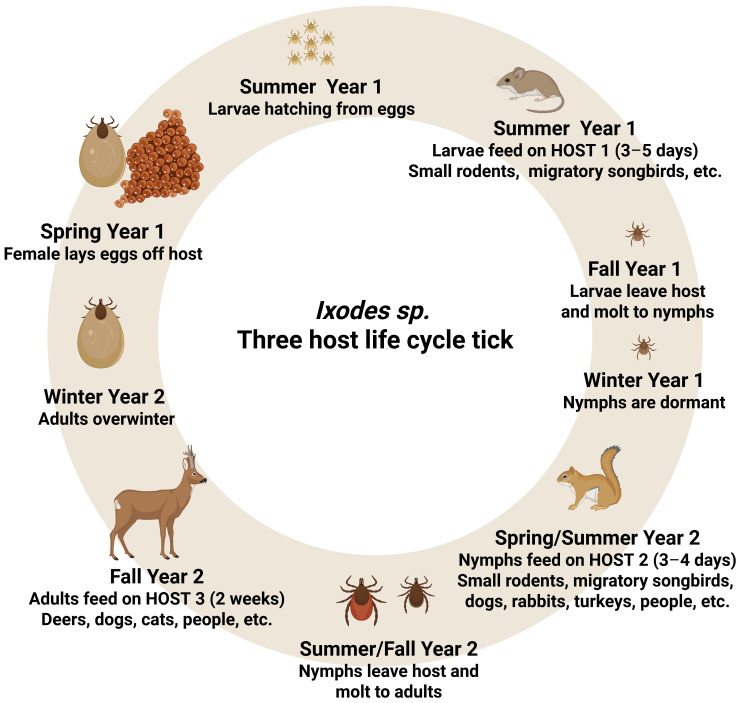
Scheme of three-host life cycle of *Ixodes* ticks genus. Created in BioRender. Szalata, M. (2026) https://BioRender.com/8qfghhx.

The tick initially feeds as a larva, then as a nymph, and finally as an adult, with each generation lasting 1–2 years, or even 3 to 6 years [11]. The life cycle of hard ticks varies depending on the number of host changes: three feeding and molting stages are required; young ticks molt on or off the host. Ticks are classified as single-, double-, or triple-host ticks. Three-host ticks include most *Ixodidae* species, such as *Dermacentor variabilis* (Say 1821), *Ixodes ricinus*, and *Ixodes scapularis* (Figure 1). After feeding in the larval and nymphal stages, they detach from the host, molt on the ground, and search for another host. Adult females feed on the host, mate, and after feeding, they drop to the ground and lay a batch of eggs, which can range from 2000 to 3000 eggs in quantity [11]. Two-host ticks, such as *Rhipicephalus bursa* (Canestrini & Fanzago 1878), feed in the larval stage and molt up to the nymphal stage on sheep. Once the nymphs are full, they detach, drop to the ground, and molt into adults. Adults search for a new host, mate, and feed until they are finished; then, they detach and drop to the ground to lay eggs [22]. In the case of single-host ticks, such as *Rhipicephalus* (*Boophilus*) *microplus* (Canestrini 1888), larvae and nymphs feed and molt on cattle; only fed and fertilized females drop from the host to the ground to lay eggs. All developmental stages of the winter tick *Dermacentor albipictus* (Packard 1869) take place on one host: elk *Cervus canadensis* (Erxleben 1777), white-tailed deer *Odocoileus virginianus* (Zimmermann, 1780), mule deer *Odocoileus hemionus* (Rafinesque 1817), caribou *Rangifer tarandus* (Linnaeus 1758), or, for the majority, moose *Alces alces* (Linnaeus 1758) [23].

## 4. Tick Habitat

We can distinguish ticks that live in nests and sheltered habitats (nidicolous ticks), including most soft ticks and a few hard ticks, compared to ticks that live in a field (non-nidicolous ticks). Free-living hard ticks inhabit forests, savannas, scrublands, heathlands, pastures, meadows, bushes, and even deserts. Their elevation above the ground depends on humidity and the host’s needs. The main tick in Europe is *Ixodes ricinus*, which is mainly found in oak and beech forests, scrub, sheep pastures on uneven hilly terrain, and in damp pastures in valleys (Great Britain) [11]. Due to their low mobility, ticks tend to gather around paths frequented by animals. Young *I. ricinus* individuals are found in areas with a high density of host populations, mainly forest mice and voles. They are increasingly found in urban areas in parks, private gardens, and industrial areas, and it is assumed that the risk of infection with certain pathogens is comparable to that in forest habitats.

Ticks demonstrate different host-finding strategies, including passive (quest) and active (hunt) methods. The passive form involves waiting for a host to appear along animal paths (e.g., *Ixodes ricinus*, *Ixodes scapularis* and *Dermacentor variabilis*) or crawling into nests. *Ixodes ricinus*, depending on the stage of development, occurs at different heights above the ground: larvae exist mainly on grass up to 30 cm high, nymphs live on green plants and shrubs up to 1 m high, and adults can be found in trees 1.5 to 2.0 m high. The active hunting strategy mainly involves reaching the host (e.g., camel tick *Hyalomma dromedarii*, Koch 1844). The host’s presence is recognized by vibration, heat, shade, odor, and carbon dioxide. The vertical distribution of hunting ticks is associated with specific groups of hosts; the presence of adults near the ends of grass or on bushes allows them to feed on large animals, while the presence of larvae and nymphs close to the ground allows contact with small mammals, birds, or lizards. The occurrence and abundance of ticks are determined not only by the availability of hosts but also by suitable microclimatic conditions, including air saturation with water vapor, poor air circulation, and low daily temperature amplitude. Ticks do not show a preference for specific plant species. However, the requirements of available hosts may coincide with the environmental requirements of ticks, e.g., temperature, humidity, and availability of plant food.

**Figure 2:**
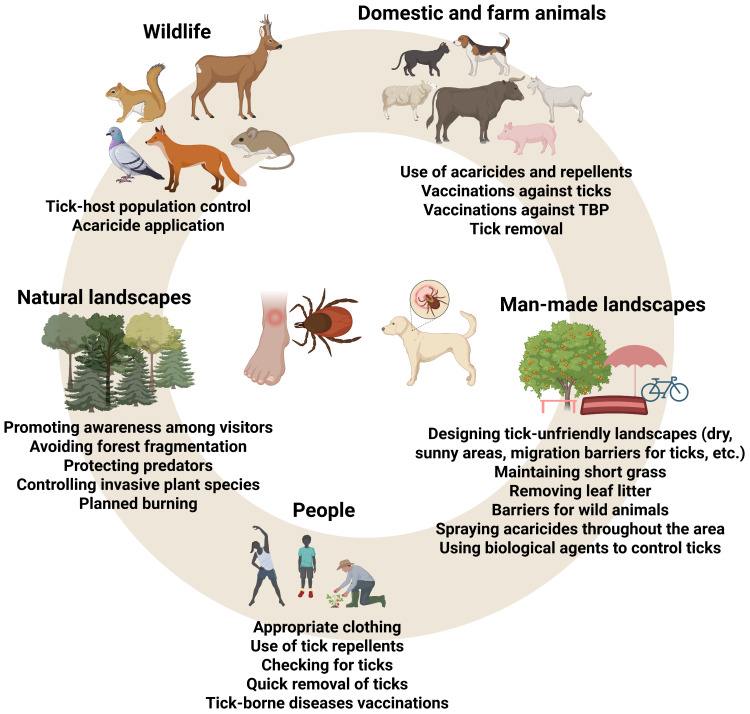
Examples of strategies reducing or eliminating risk associated with tick-borne diseases [26,27]. Created in BioRender. Szalata, M. (2026) https://BioRender.com/e1c4d8c.

Biological solutions, including plants, the use of natural predators, host control, entomopathogenic fungi, physical interventions, and habitat modification, are becoming increasingly effective methods for controlling tick populations and are being incorporated into sustainable and environmentally friendly agricultural practices (Figure 2). Integrated Pest Management (IPM) requires long-term solutions [24,25].

Urban green spaces are becoming new, often unexpected, habitats for ticks. Recreational use of urban green spaces, in addition to health benefits, is associated with an increased risk of contact with ticks, which are vectors of various diseases. Research conducted in Stockholm, Sweden, revealed high densities of *Ixodes ricinus* and taiga tick *Ixodes persulcatus* (Schulze 1930) in five selected microhabitats. This, combined with the presence of *Borrelia burgdorferi sensu lato* (Johnson et al. 1984 emend. Baranton et al. 1992) and *Anaplasma phagocytophilum* (Foggie 1949, Dumler et al. 2001), increases the risk of human exposure to tick-borne diseases, particularly in the active urban green spaces. Researchers have emphasized the need for public health campaigns to reduce the risk of tick-borne diseases [27]. Domestic dogs, as carriers of the Marsh tick *Dermacentor reticulatus* (Fabricius 1794), may pose a risk of introducing ticks into homes. Domestic conditions favor the development of tick stages, and female ticks feeding on dogs demonstrate high reproductive efficiency. Ticks can be transmitted between pets in urban areas, but they can also use humans as hosts [28].

In Europe, the most common and important species from a medical perspective are wood ticks such as *Ixodes ricinus*, brown dog ticks such as *Rhipicephalus sanguineus* (Latreille 1806) and *Hyalomma marginatum* (Koch 1844), and Ornate sheep ticks such as *Dermacentor marginatus* (Sulzer 1776), and *Dermacentor reticulatus* [29,30,31]. In Africa, significant problems are connected with the Tropical bont tick *Amblyomma variegatum* (Fabricius 1794), *Hyalomma* spp. (Koch 1844), Brown ear tick *Rhipicephalus appendiculatus* (Neumann 1901), and Eyeless tampan *Ornithodoros moubata* (Murray 1877), and this data is underestimated. In Asia, the most commonly recorded species are as follows: *Dermacentor reticulatus*, *Haemaphysalis flava* (Neumann 1897), Bush tick *Haemaphysalis longicornis* (Neumann 1901), *Haemaphysalis qinghaiensis* (Teng 1980), *Haemaphysalis spinigera* (Neumann 1897), *Hyalomma* spp., *Ixodes persulcatus*, Asiatic cattle tick *Rhipicephalus microplus* (Canestrini 1888), and *Rhipicephalus sanguineus*. In Australia and New Zealand, the following ticks are found: *Haemaphysalis longicornis*, Australian paralysis tick *Ixodes holocyclus* (Neumann 1899), and *Rhipicephalus microplus*. In North America, tick populations include *Ixodes scapularis*, Lone star tick *Amblyomma americanum* (Linnaeus 1758), *Dermacentor variabilis,* and Western black-legged tick *Ixodes pacificus* (Cooley & Kohls 1943). Finally, in South America, the following species are common: Cayenne tick *Amblyomma cajennense* (Fabricius 1787), *Amblyomma variegatum*, *Rhipicephalus microplus*, and *Rhipicephalus sanguineus* [31].

According to the Italian EpiCentro–Epidemiology for public health, 36 tick species are present in Italy; these include mainly hard ticks like *Ixodes* (Latreille 1795), *Rhipicephalus* (Koch 1844), *Hyalomma*, *Haemaphysalis* (Koch 1844), *Dermacentor* (Koch 1844), and soft ticks such as *Argas* (Latreille 1795) (Pigeon tick *Argas reflexus*, Fabricius 1794) and *Ornithodoros* (Koch 1837) [29]. In the United Kingdom, according to data from the UK Health Security Agency from March 2025, 20 tick species have been observed, mainly *Ixodes* spp., *Dermacentor reticulatus*, Red sheep tick *Haemaphysalis punctata* (Canestrini & Fanzago 1878), *Carios maritimus* (Vermeil & Marguet 1967), *Argas reflexus*, Blyborough tick *Argas vespertilionis* (Latreille 1796), and imported species like *Rhipicephalus sanguineus* and *Hyalomma marginatum* [30]. In Germany between 2018 and 2020, during planned questing tick collection campaigns (in 83 locations), over 50,000 individuals in various stages of development were collected, most of which represented the species *Ixodes ricinus* (98.6%). The second most common tick was *Dermacentor reticulatus* (1.3%), and a few specimens of the species *Ixodes frontalis* (Panzer 1798), *Ixodes festai* (Tonelli-Rondelli 1926), and *Dermacentor marginatus* were also collected [32]. The highest density of ticks that may be a source of exposure to pathogens was the *Borrelia* (Swellengrebel 1907) and *Anaplasma phagocytophilum* spp. observed in coastal areas and mid-elevation mountain ranges.

The European Centre for Disease Prevention and Control, an agency of the European Union, mainly focuses on *Ixodes ricinus* and *Hyalomma marginatum* as disease vectors and tracking the occurrence of *Dermacentor reticulatus*, *Hyalomma lusitanicum* (Koch 1844), *Hyalomma marginatum*, *Ixodes persulcatus*, *Ixodes ricinus*, *Ornithodoros erraticus* (other name *Carios erraticus*, Lucas, 1849), and *Rhipicephalus sanguineus* [33].

The U.S. Centers for Disease Control and Prevention (CDC) is gathering information about the American dog tick *Dermacentor variabilis* (Say 1821), Western United States tick *Dermacentor similis* (Lado, Glon, and Klompen 2021), *Haemaphysalis longicornis, Ixodes scapularis*, *Rhipicephalus sanguineus*, Gulf Coast tick *Amblyomma maculatum* (Koch 1844), *Amblyomma americanum*, Rocky Mountain wood tick *Dermacentor andersoni* (Stiles 1908), and *Ixodes pacificus* [34].

In Central Europe 19 species of ticks (*Ixodida*) are recognized: *Argas reflexus*, *Argas polonicus* (Siuda, Hoogstraal, Clifford & Wassef 1979), Blyborough tick *Carios vespertilionis* (Latreille 1796), Shrew tick *Ixodes trianguliceps* (Birula 1895), Tree hole tick *Ixodes arboricola* (Schulze & Schlottke 1930), *Ixodes crenulatus* (Koch 1844), Hedgehog tick *Ixodes hexagonus* (Leach 1815), Sand Martin tick *Ixodes lividus* (Koch 1844), *Ixodes rugicollis* (Schulze & Schlottke 1930), Northern bird tick *Ixodes caledonicus* (Nuttall 1910), Passerine tick *Ixodes frontalis*, *Ixodes simplex* (Neumann 1906), Long legged bat tick *Ixodes vespertilionis* (Koch 1844), Marsh tick *Ixodes apronophorus* (Schulze 1924), *Ixodes persulcatus*, *Ixodes ricinus*, *Haemaphysalis punctata*, Bush Tick *Haemaphysalis concinna* (Koch 1844) and *Dermacentor reticulatus*. The most common species with the greatest medical and veterinary importance in Europe is the common tick *Ixodes ricinus*; however, the meadow tick *Dermacentor reticulatus* is also expanding its range of occurrence and is becoming increasingly important in the epidemiology of transmissible diseases.

Alien tick species are occasionally recorded in Europe; these are mainly transmitted through the movement of exotic animals. These include the Tuatara tick *Amblyomma sphenodonti* (Dumbleton 1943), *Amblyomma exornatum* (Koch 1844), *Amblyomma flavomaculatum* (Lucas 1846), *Amblyomma latum* (Koch 1844), Hard tick *Amblyomma nuttalli* (Dönitz 1909), *Amblyomma quadricavum* (Schulze 1941), *Amblyomma transversal* (Lucas 1845), *Amblyomma varanense* (Supino 1897), *Amblyomma* spp., *Dermacentor marginatus*, tick *Hyalomma aegyptium* (Linnaeus 1758), *Hyalomma marginatum*, *Ixodes eldaricus* (Dzhaparidze 1950), *Ixodes festai*, *Rhipicephalus rossicus* (Yakimov & Kol-Yakimova 1911) and *Rhipicephalus sanguineus* [35,36,37,38].

## 5. Tick Genome

Deciphering the *Ixodes scapularis* genome took 10 years due to significant difficulties in interpreting its gene structure. The project was carried out by an international research team (93 authors from 46 centers) led by scientists from Purdue University (USA). The research proved more difficult than with malaria-carrying mosquitoes, despite the ticks themselves being considerably less dangerous. One of the greatest challenges was the complexity of the tick genome—it is one of the largest arthropod genomes sequenced to date. Approximately 70% of the genome consists of repetitive genes, which are often mutated. It is possible that individual copies of the same gene function slightly differently, facilitating the arachnid’s survival [39,40]. Similarly, the hard tick *Ixodes ricinus* is characterized by a large genome containing many repeated elements. Recent studies of the hard tick *Ixodes ricinus* have confirmed the presence of numerous transposable elements (a minimum of 69%, two sets of the haploid genome, approximately 2.15 Gbp in size). Compared to *Ixodes scapularis*, it has been suggested that the diversity of transposable elements is responsible for the evolution of the tick genome [41].

Ticks transmit more diseases and parasites than other arthropods. Thousands of people and animals die each year from diseases such as anaplasmosis, babesiosis, Powassan virus, and encephalitis. This research focuses on both genes and proteins that facilitate parasitism and disease transmission. This should aid in the development of new drugs and vaccines. Unique aspects of tick biology have been identified. Saliva contains thousands of substances (hundreds in mosquitoes) that have antibacterial, analgesic, anticoagulant, and immunosuppressive properties, allowing ticks to suck blood without the host’s knowledge. Genes have been identified that allow the tick to produce a new exoskeleton (which can enlarge up to 100-fold after feeding), as well as genes that facilitate feeding on blood containing high levels of iron (excess iron is toxic). Ticks also possess enzymes that can inactivate insecticides. While *Ixodes scapularis* ticks found in the Southern and Northern United States do not differ genetically enough to be considered separate species, more cases of Lyme disease occur in the northern regions; it is likely that some characteristic of the ticks there plays a role in disease transmission [42].

## 6. Factors Influencing Tick Exposure

### 6.1. Epidemiology

Ticks feed when temperatures are above freezing and humidity is high; therefore, the peak incidence of tick-borne diseases occurs from May to September. Ticks transmit diseases such as Lyme disease and tick-borne encephalitis.

Central Europe is an endemic area for Lyme disease. While the number of new cases has begun to rise after the COVID-19 pandemic, with an increase in outdoor activity, it has not yet reached the pre-2020 level. There were over 17,000 cases in 2022 compared to over 20,000 cases pre-2020, giving an incidence of 45.9 in 2021, compared to an average incidence of 53.7 per 100,000 in the total population in 2022 [43]. Between 2015 and 2023, an average of 132,000 confirmed Lyme disease cases were reported annually in Europe (29 countries). The highest number of cases, exceeding 100 cases per 100,000 inhabitants per year, was observed in Estonia, Finland, and Slovenia. An increase in incidence occurred throughout Europe, with particular attention paid to northern, eastern, and western Europe [44]. In the case of Lyme disease, correct diagnosis is very important; thus, Lyme disease is associated with overdiagnosis, and the introduction of ineffective antibiotic therapy has been reported in upwards of 45–75% of such cases [45]. Correct diagnosis is challenging as similar symptoms are associated with rheumatic, musculoskeletal, and neurological diseases [46,47].

Cases of tick-borne encephalitis (TBE) occur in Europe and Asia, with the Central European virus being the most common in Europe. The Siberian virus type, present in Russia and the Baltic states, is not observed. According to the CDC, tick-borne encephalitis ranges from western and northern Europe to North and East Asia but is not present in the United States. TBE is endemic in Belarus, the Czech Republic, Estonia, Latvia, Lithuania, Poland, Slovenia, Sweden, and Switzerland. Endemic foci are found in Austria, Finland, Slovakia, and the United Kingdom. TBE has a variable regional distribution in China, Croatia, Denmark, France, Germany, Hungary, Italy, Kazakhstan, Kyrgyzstan, Mongolia, Norway, Russia, and Ukraine. In Europe, there are over 10,000 new cases per year. At the same time, an increase in the number of people vaccinated against TBE was observed, especially among people who spend a lot of time in forests and wooded areas. Therefore, extending the scope of vaccinations is mandatory, especially for people who work professionally in forests. It should also be remembered that it is possible to become infected with the virus through the digestive tract by consuming unpasteurized milk from infected animals, mainly goats, though these cases are rare. Tick-borne encephalitis (TBE) rates of more than 5 cases per 100,000 people were reported between 2020 and 2023 in Lithuania, Latvia, Estonia, the Czech Republic, and Slovenia. A similarly high rate was observed among unvaccinated populations in Sweden and Austria [48]. Further studies, based on the analysis of patients treated between 2018 and 2022 in selected hospital wards, indicated that TBE was only confirmed in some cases showing clinical symptoms of this disease. TBE was confirmed in 124 out of 766 patients, none of whom had previously been vaccinated against TBE [49]. Unlike Lyme disease, TBE is detected less frequently, leading to an underestimation of the actual number of cases.

In response to growing interest in the detection and treatment of diseases transmitted by ticks, among other concerns, the EU Reference Laboratory for Public Health on “High-risk, emerging and zoonotic bacterial pathogens” (EURL-PH-HEZB, Grant Agreement: 101194786) began operating in 2025 as part of the EU Reference Laboratory for Public Health (EURLs). The consortium, coordinated by the German Robert Koch Institute, collaborates with partners such as the Bundeswehr Institute of Microbiology in Germany, the Swedish Folkhälsomyndigheten, and the Instituto Nacional de Saúde Doutor Ricardo Jorge in Portugal. The aim is to strengthen the capacity of national specialist laboratories and to support the harmonized surveillance, notification, and reporting of diseases or causative bacterial agents in accordance with the recommendations of the European Center for Disease Prevention and Control (ECDC). The consortium deals with emerging and zoonotic bacterial pathogens, including *Bacillus anthracis* (Cohn 1872, anthrax), *Yersinia pestis* ((Lehmann & Neumann 1896) van Loghem 1944, plague), *Francisella tularensis* ((McCoy and Chapin 1912), Dorofe’ev 1947, tularemia), *Brucella* spp. (Meyer and Shaw 1920, brucellosis), *Burkholderia mallei* ((Zopf 1885) Yabuuchi et al. 1993, glanders), *Burkholderia pseudomallei* (melioidosis), *Coxiella burnetii* ((Derrick 1939) Philip 1948, Q fever), *Rickettsia* spp. (da Rocha-Lima 1916, rickettsiosis), *Borrelia* spp. (Lyme borreliosis), and *Leptospira* spp. (Noguchi 1917 non Swainson 1840 non Boucot, Johnson & Staton 1964, leptospirosis).

Many of these pathogens are transmitted by ticks [50]; hence, numerous studies are being conducted to detect them. For example, two species of ticks, *Dermacentor reticulatus* (339 individuals) and *Ixodes ricinus* (353 individuals), collected between 2021 and 2024 in Germany, were analyzed [51]. The authors focused on the detection of *Francisella tularensis* subsp. *holarctica*, *Francisella*-like endosymbionts, *Rickettsia* spp., *Borrelia burgdorferi* sensu lato complex (Johnson et al. 1984, emend. Baranton et al. 1992), *Borrelia miyamotoi* (Fukunaga et al. 1995), *Anaplasma phagocytophilum*, *Ehrlichia* spp. (Moshkovski 1945 (Approved Lists 1980), emend. Dumler et al. 2001), *Coxiella burnetii*, *Bartonella* spp. (Strong et al. 1915, Approved Lists 1980), *Babesia* spp. (Starcovici 1893), and the tick-borne encephalitis virus, with particular emphasis on *Francisella tularensis* subsp. *holarctica*, which is the most important subspecies causing tularemia in Europe and Germany. Depending on the tick species, the presence of mainly *Francisella*-like endosymbionts (18–97%), *Rickettsia* spp. (32–74%) and *B. burgdorferi* (0–16%) was demonstrated. No *Coxiella burnetii*, *Ehrlichia* spp., or *Bartonella* spp. were detected, and other pathogens occurred at low frequencies in ticks; moreover, in many cases, ticks were carriers of two or more pathogens [51]. The occurrence of co-infections can lead to difficulties in identifying and treating patients, as this possibility is not taken into account when choosing treatment methods [52,53].

Studies covering 1619 *Ixodes ricinus* ticks at various stages of development collected in 2012 in Central Europe indicated the presence of *Anaplasma phagocytophilum* (0.54%) and *Rickettsia helvetica* (Beati et al. 1993) (3.69%), while no evidence of *Francisella* spp. (Dorofe’ev 1947 (Approved Lists 1980) emend. Huber et al. 2010) was detected [54]. Subsequent studies covered 141 ticks (104 *Ixodes ricinus* and 37 *Dermacentor reticulatus*) collected in the spring of 2018 [49]. The presence of *Borrelia* spp. (14.9%), *Babesia* spp. (10.6%) and *Rickettsia* spp. (17.7%) was indicated, while *Anaplasma phagocytophilum* was not detected. The authors observed differences in the occurrence of individual species of pathogens in both tick species: while some were present exclusively in *I. ricinus* (*Borrelia afzelii* (Canica et al. 1994), *Borrelia garinii* (Baranton et al. 1992), and *Borrelia burgdorferi* s.s., *Rickettsia helvetica*), others were found only in *D. reticulatus* (*Babesia microti* (Franca, 1912) and *Rickettsia raoultii* (Mediannikov et al. 2008)) [55].

Analysis of tick-borne pathogens is widely conducted; they show specificity depending on the region and tick species, and examples can be developed further [56,57,58].

### 6.2. Prevention

Appropriate clothing (light)—long pants, a long-sleeved shirt tucked into the pants, and closed shoes—makes it more difficult for ticks to reach the skin, and easier for the wearer to spot ticks on their outfit. Upon return from a potentially tick-infested area, one should shake their clothes outside, take a shower, and inspect their skin. If a tick is found, it should be removed by grasping it as close to the skin as possible with tweezers, a tick card, or another tool available at the pharmacy. It is important to use a single steady motion, move perpendicular to the skin to remove the tick, and disinfect the skin afterwards. It is best to remove the tick within 24 h of attachment to prevent tick-borne pathogens from entering the body. Skin-based repellents are also an effective countermeasure, but they can cause allergic reactions [43,46].

### 6.3. Vaccination

The increasing incidence of Lyme disease, tick-borne encephalitis (TBE), and other tick-borne diseases such as babesiosis, ehrlichiosis, rickettsiosis, and anaplasmosis is becoming a growing public health concern. Current strategies for preventing tick bites are insufficient, which is why vaccines are a promising preventive measure.

Due to the growing threat of tick-borne diseases, attention is being increasingly paid to the possibility of preventing their development. One strategy, in addition to limiting contact with tick habitats, using repellents, and removing ticks within 24 h of a bite, is protective vaccination. Vaccines against tick-borne encephalitis (TBE) are currently available on the market and are recommended for children aged 13 to 15 months and for adults, especially in the case of additional risk factors, e.g., medical, occupational, or lifestyle-related factors [59]. In Europe, the following vaccines based on inactivated viruses have been approved for a number of years: Encepur Adults FSME-Immun for adults and Encepur K and FSME-Immun Junior for children [60]. A vaccine against Lyme disease is in an advanced stage of clinical trials. From 1998, the LYMErix vaccine was approved by the FDA and made available in the United States. It acted against Lyme disease based on the OspA antigen. However, the product was withdrawn in 2002; rather than safety concerns, this was due to the reluctance of people at risk of Lyme disease to use it. No attempt was made to commercialize another, improved vaccine; however, the need to develop a multivalent vaccine that is protective against different *Borrelia* serotypes has been pointed out [59].

### 6.4. Emerging Issues

Increasing attention is being paid to ticks as potential vectors of various pathogens [52]. It should be noted that some of these pathogens are endemic, but due to the extension of the tick feeding period and their range as a result of climate change, human migration, human activities, their ability to spread quickly, and the plasticity of the tick genome, they may pose an increasing problem [56,57,61]. Ticks and the diseases they transmit are of particular interest to professional groups that use forest areas extensively, including forestry services and military services, due to the numerous threats associated with tick bites [58]. This enables the implementation of preventive and, if necessary, therapeutic measures. One of the authors (RS) was involved in research on tick-borne diseases at the Bundeswehr Institute of Microbiology in Munich even before the pandemic.

Research suggests that alcohol consumption may increase a person’s exposure to ticks and mosquitoes, but this effect can be explained by an alteration in behavior and movements [62]. Recent tests also indicate that ticks *Ixodes ricinus*, especially those carrying dangerous pathogens, respond positively to electromagnetic radiation [63,64] and electromagnetic fields. The reaction to electromagnetic radiation appears to vary among different groups of ticks and requires further research [65].

*Ixodes ricinus* tick larvae are usually carriers of babesiosis—*Babesia divergens* (M’Fadyean & Stockman, 1911) is transmitted by female ticks through the ovaries to the eggs, i.e., transovarial transmission, which means that the mother passes it on to her offspring [66]. In the case of another pathogen belonging to the genus *Babesia*, such as *Babesia microti*, which is transmitted by *Ixodes scapularis* in North America, transovarial transmission does not occur. This may be due to the significant genetic distinctiveness of *B. microti* [31].

## 7. Tick-Borne Diseases

Various tick-borne diseases in the Northern Hemisphere include Lyme disease, Q fever (though this is rare and more often transmitted through contaminated feces), Colorado tick fever, Rocky Mountain spotted fever, African tick-borne fever, Crimean–Congo hemorrhagic fever, tularemia, relapsing fever, babesiosis, ehrlichiosis (anaplasmosis), tick-borne encephalitis, and bovine anaplasmosis. In Europe, the most common infections are Lyme borreliosis and tick-borne encephalitis (a virus from the *Flaviviridae* family), sporadic Q fever (goat fever, a Gram-negative bacilli of the *Coxiella* genus (Philip 1943, Philip 1948, Approved Lists 1980), tularemia (rabbit fever, *Francisella tularensis*), bartonellosis (cat scratch disease, *Bartonella* genus), babesiosis (protozoa of the *Babesia* genus), ehrlichiosis (anaplasmosis, Gram-negative bacteria *Anaplasma phagocytophilum*), and spotted fever rickettsioses (Gram-negative bacteria) [42].

Lyme borreliosis, also known as tick-borne spirochetosis or Lyme disease, is a multisystem zoonotic disease with autoimmune features, caused by spirochetes of the *Borrelia* genus, which are transmitted to humans via ticks of the *Ixodes* genus. The name comes from the towns of New Lyme and Old Lyme, Connecticut, in the United States, where the first cases of arthritis among adolescents with tick bites were described in 1977. Diagnosis of Lyme disease became possible after the isolation of the *Borrelia burgdorferi* bacterium, made by Willy Burgdorfer, an American bacteriologist and parasitologist of Swiss descent, in 1981. The diagnosis and treatment of Lyme disease require the presence of characteristic clinical symptoms confirmed by serological testing, with the exception of erythema migrans, which does not require any testing. Currently, a two-step diagnostic algorithm is recommended, which does not include testing the tick [46,67,68]. In the first step, a quantitative analysis of antibodies specific to *Borrelia burgdorferi*, a bacterium transmitted by ticks, is performed on the patient’s blood serum (the test is very sensitive). In the case of a positive or inconclusive result, a second test is performed using the Western blot method, which ensures the specificity of the test. Negative results in the first stage using tests such as ELISA, Enzyme-Linked Immunosorbent Assay, CLIA Chemiluminescence ImmunoAssay, and MMIA Multiplexed Microbead ImmunoAssay exclude Lyme disease and prevent the need for further testing [46].

Tick-borne encephalitis is an inflammatory disease of the central nervous system caused by a virus belonging to the *Flaviviridae* family. Q fever (goat fever) is a bacterial infectious disease of cattle, sheep, and goats caused by Gram-negative bacilli of the genus *Coxiella*. Humans become infected by inhaling dust contaminated with the feces, urine, or milk of infected animals, or by ticks. Tularemia (rabbit fever) is a bacterial disease caused by the bacterium *Francisella tularensis*, which is transmitted by ticks and deer flies. Typical symptoms include fever, chills, headache, diarrhea, vomiting, muscle and joint pain, and sometimes a macular or maculopapular rash. Bartonellosis (also known as cat scratch disease) is an infectious disease caused by bacteria of the genus *Bartonella*. It is usually transmitted to humans through scratches from animals (primarily young cats), and occasionally through tick bites. Babesiosis is a disease caused by protozoa of the genus *Babesia*. Their hosts are typically domestic and wild animals, such as rodents, cattle, dogs, and cats, and their vectors are ticks. Symptoms are nonspecific: fever with chills, fatigue, headaches and muscle pain, increased sweating, malaise, reluctance to eat, vomiting, and abdominal pain. In more severe cases, jaundice, dark urine, respiratory distress, stiff neck, coma, and kidney and liver failure may occur. Ehrlichiosis is an infectious disease caused by the Gram-negative bacterium *Anaplasma phagocytophilum*. The most common symptoms include high fever, chills, headaches, muscle and joint pain, and rash. Rickettsioses, also known as spotted fevers, are diseases caused by rickettsiae. They mainly reside in mammals and are transmitted by arthropods (ticks, lice, fleas, mites). Rickettsiae cause vasculitis, primarily of the small arteries and capillaries [69].

## 8. Protection Methods

Ticks occur naturally in the environment, providing a food source for spiders, ants, beetles, and many birds, including domestic birds such as guinea fowl, and small mammals such as shrews and mice. It can, therefore, be assumed that, as part of the food chain, ticks help in the circulation of nutrients in ecosystems, including the redistribution of nitrogen, phosphorus, and carbon into the soil, and can be used to monitor the health of ecosystems [17,70]. Through feeding, they can affect the populations of natural hosts, such as deer, rodents, birds, and reptiles, influencing their numbers and, thus, indirectly affecting the environment in which they live [71,72]. It is assumed that the diversity of host populations can influence environmental health and biodiversity [73,74]. The diversity of the tick population then becomes a kind of bioindicator of the health of the ecosystem. In addition, maintaining leaf litter for tick habitats can improve soil health in degraded areas. However, it should be remembered that anthropogenic human impact can disrupt this balance, as seen through the effects of climate change (causing temperature increases and a reduction in natural forest areas), which leaves animals vulnerable, more susceptible to tick-borne diseases, and causes the extinction of animal populations [75]. An alternative approach is to control tick populations by promoting habitats for birds that feed on ticks. A sustainable approach for humans may include preventing tick bites by wearing appropriate clothing and using repellents, checking the body after returning home, and limiting contact with ticks when they are most active. This approach is important because it does not disturb the ecological balance of the environment [76]. In natural environments, a method of controlling tick populations is to reduce vegetation density in order to limit tick habitats. Another approach involves increasing sunlight exposure and reducing humidity, thereby limiting the conditions in which ticks can live. Another important strategy is the access of wild animals and habitats to human property by using fencing and appropriate plant selection.

Increasingly, attention is being paid to the use of plant acaricides and repellents, with particular emphasis on minimally processed products, which can provide a synergistic effect while reducing production and application costs and limiting negative environmental impact. There is emerging interest in using essential oils or extracts from the entire plant or selected parts (e.g., leaves, flowers, roots, and fruits). Nanoparticles, nanomaterials, and photosensitizers are also being considered, but the possibility of selective action on target ticks should be considered. Chemical acaricides can also be reduced through the use of entomopathogenic fungi [77]. Plants from several major families have traditionally been used to reduce contact with ticks and treat diseases caused by them, but this does not cover all possibilities. Recently, in addition to traditionally used plants, new species have also been investigated [25,78,79,80]. We can protect ourselves against ticks and the diseases they transmit through appropriate use of plants and the application of biotechnology [81]. Specific plants can serve as a natural repellent against ticks in backyards. These plants can also be used as a source of bioactive substances that repel ticks, which could then be formulated into bio-repellents. To achieve this, the choice of plant, substance, and extraction and application methods is crucial [82]. The following plants and sprays are suitable to repel ticks: Common Tansy (*Tanacetum vulgare* L. 1753, family *Asteraceae* Bercht. & J.Presl, 1820), Old Tansy (*Tanacetum cinerariifolium* (Trevis.) Sch. Bip., 1844, perennial from the *Asteraceae* family, formerly *Dalmatian chrysanthemum*), Common wormwood (*Artemisia absinthium* L. 1753, family *Asteraceae*), Catmint (Faassen’s catnip, *Nepeta* × *faassenii* Bergmans ex Stearn, primary hybrid *Nepeta racemosa* Lam. 1785 and *Nepeta nepetella* L. 1759, family *Lamiaceae* Martinov 1820), Lavender (*Lavandula angustifolia* Mill. 1768, common names lavender, true lavender, English lavender, garden lavender, common lavender and narrow-leaved lavender, family *Lamiaceae*), Rosemary (*Rosmarinus officinalis* Spenn. 1835, shrub from the sage family *Lamiaceae*), Horseradish (*Armoracia rusticana* G. Gaertn., B. Mey. & Scherb. 1800, cabbage family *Brassicaceae* Burnett 1835), Sweet Flag (*Acorus calamus* L. 1753, perennial from the *Calamus*, family *Acoraceae* Martinov 1820), Onion (*Allium cepa* L. 1753, *Amaryllis* L. 1753 family *Amaryllidaceae* J. St.-Hil. 1805), Buttercup (*Ranunculus* L. 1753, family *Ranunculaceae* Juss. 1789), Wild Garlic (*Allium ursinum* L. 1753, family *Amaryllidaceae*), and *Cannabis sativa* L. 1753 (family *Cannabaceae* Martinov 1820) [83,84,85]. The following plant families are of great interest as acaricides and repellents against ticks: *Meliaceae* Juss. (*Azadirachta indica* A. Juss. 1831, *Melia azedarach* L. 1753), *Rutaceae* Juss. 1789 (citrus L. 1753, *Citrus aurantiifolia* (Christm.) Swingle 1913, *Clausena anisate* (Willd.) Hook.f. ex Benth., 1849, *Citrus sinensis* (L.) Osbeck 1765 var. *balady*; *Citrus limon* (L.) Osbeck 1765), *Amaryllidaceae* (garlic *Allium sativum* L. 1753), *Asphodelaceae* Juss. 1789 (*Aloe rupestris* Baker 1896, *Aloe ferox* Mill. 1768, *Aloe vera* (L.) Burm.f. 1768), *Cucurbitaceae* Juss. 1789 (*Citrullus colocynthis* (L.) Schrad. 1838, *Momordica charantia* L. 1753), *Calophyllaceae* J. Agardh 1858 (*Mammea americana* L. 1753), *Asteraceae* (*Achillea millefolium* L. 1753, *Artemisia absinthium*, *Artemisia herba-alba* Asso 1779, *Artemisia monosperma* Delile 1813, *Bethencourtia palmensis* Choisy 1828, *Carthamus tinctorius* L. 1753, *Chamaemelum nobile* (L.) All. 1785, *Dysphania ambrosioides* (L.) Mosyakin & Clemants 2002, former *Chenopodium ambrosioides* L. 1753, chamomile *Matricaria chamomilla* L. 1753, *Schkuhria pinnata* (Lam.) Kuntze ex Thell. 1912, *Tagetes minuta* L. 1753, *Thymus vulgaris* L. 1753, *Thymus zygis* L. 1753, *Pulicaria undulata* (L.) C. A. Mey. subsp. *undulata*), *Fabaceae* Lindl. 1836 (*Calpurnia aurea* Benth. 1837, *Lupinus albus* L. 1753), *Lamiaceae* (Rosemary, *Lavandula angustifolia*, *Lavandula pedunculata* subsp. *Atlantica* (Braun-Blanq.) Romo, *Mentha suaveolens* Ehrh. 1792, *Ocimum basilicum* L. 1753, *Ocimum americanum* L. 1755 synonim *Ocimum canum*, *Clinopodium nepeta* subsp. *spruneri* (Boiss.) Bartolucci & F. Conti 2011 synonym *Satureja calamintha*, *Satureja montana* L. 1753, *Satureja thymbra* L. 1753, *Vitex negundo* L. 1753), *Solanaceae* Juss. 1789 (*Nicotiana tabacum* L. 1753, *Withania somnifera* (L.) Dunal 1852, *Solanum trilobatum* L. 1753) and *Verbenaceae* J. St.-Hil. 1805 (*Lippia javanica* (Burm.f.) Spreng. 1825), and many others [78,79,80].

Acaricides can also be used to chemically control ticks. In many cultures, natural plant-based remedies are also used to help control ticks and treat tick-borne diseases in livestock [80]. The Food and Agriculture Organization of the United Nations aims to identify strategies for controlling ticks in cattle [86]. Having access to reliable resources is also important [87].

## 9. Conclusions

Ticks are vectors of a wide range of pathogens of global public health and veterinary importance (including viruses, bacteria, and protozoa). Changes to environments, such as climate change, are promoting their spread. Further research is required on tick biology and ecology, tick–host–pathogen interactions, the circulation of tick-borne pathogens in natural hotspots, and the epidemiology of these diseases. Their complex life cycles and sophisticated feeding strategies also pose challenges. The small size of ticks, depending on their developmental stage, makes rapid detection of feeding individuals difficult. There are no fully implemented algorithms for diagnosing tick-borne pathogens, and the co-occurrence of different pathogens within a single individual is problematic. Limitations in tick research include a lack of comprehensive diagnostics, a significant underestimation of tick-borne disease cases due to nonspecific symptoms that are often misdiagnosed as other diseases, and a lack of comprehensive monitoring. Methods to control tick populations have numerous limitations, including the use of chemicals (toxicity, cost, and resistance) and biological agents (environmental factors such as low humidity, high temperature, and UV radiation). The tick development cycle, which relies on multiple hosts, also makes tick control difficult. New tick control strategies are being explored, focusing on the use of natural plant products. Vaccines are also problematic due to their ineffectiveness. The development of effective vaccines is limited by the identification of the target antigen. Modifying the tick microbiome and analyzing the composition and properties of saliva may be a potential strategy [88,89,90].

The question remains: How can we protect ourselves from ticks? Ticks feed when the temperature exceeds zero degrees and the humidity is high. Most tick-borne diseases are transmitted between March and November. Appropriate (light-colored) clothing, such as long pants, a long-sleeved shirt tucked into pants, and shoes, makes it easier to spot ticks. Ticks can be removed by grasping them as close to the skin as possible with tweezers, a special card, or another tool available at a pharmacy. It is best to remove ticks within 24 h of attachment to prevent tick-borne pathogens from entering the body. Skin repellents can also be used, but they may cause allergic reactions. Protection against ticks and the diseases they carry can be achieved through the appropriate use of plants and the development of biotechnology. It is important to choose plants that are native to the environment, as they will contain sources of bioactive substances that repel ticks (making it important to consider the choice of plant, substance, method of extraction, and form of application). Finally, individuals should remember to walk on the sunny side of a street, as Louis Armstrong suggests: “Take your coat and hat. Leave your worries at the door. Just take a walk on the sunny side of the street…” In doing so, ticks will not be happy [91].

## Data Availability

No new data were created or analyzed in this study. Data sharing is not applicable to this article.
